# Supplementary material for: High-dimensional regression in practice: an empirical study of finite-sample prediction, variable selection and ranking
Source: Stat Comput. 2019 Dec 19;30(3):697–719. doi: 10.1007/s11222-019-09914-9 (PMC7026376; doi:10.1007/s11222-019-09914-9)
Supplement: Supplementary file 1 — Supplementary material 1 (pdf 541 KB) [file 11222_2019_9914_MOESM1_ESM.pdf]

High-dimensional regression in practice: an empirical study of finite-sample prediction, variable selection and ranking

Supplementary Material

Fan Wang[1], Sach Mukherjee[2], Sylvia Richardson[1] and Steven M. Hill[1]

1. MRC Biostatistics Unit, University of Cambridge, Cambridge, UK
2. German Centre for Neurodegenerative Diseases (DZNE), Bonn, Germany

Supplementary Figures

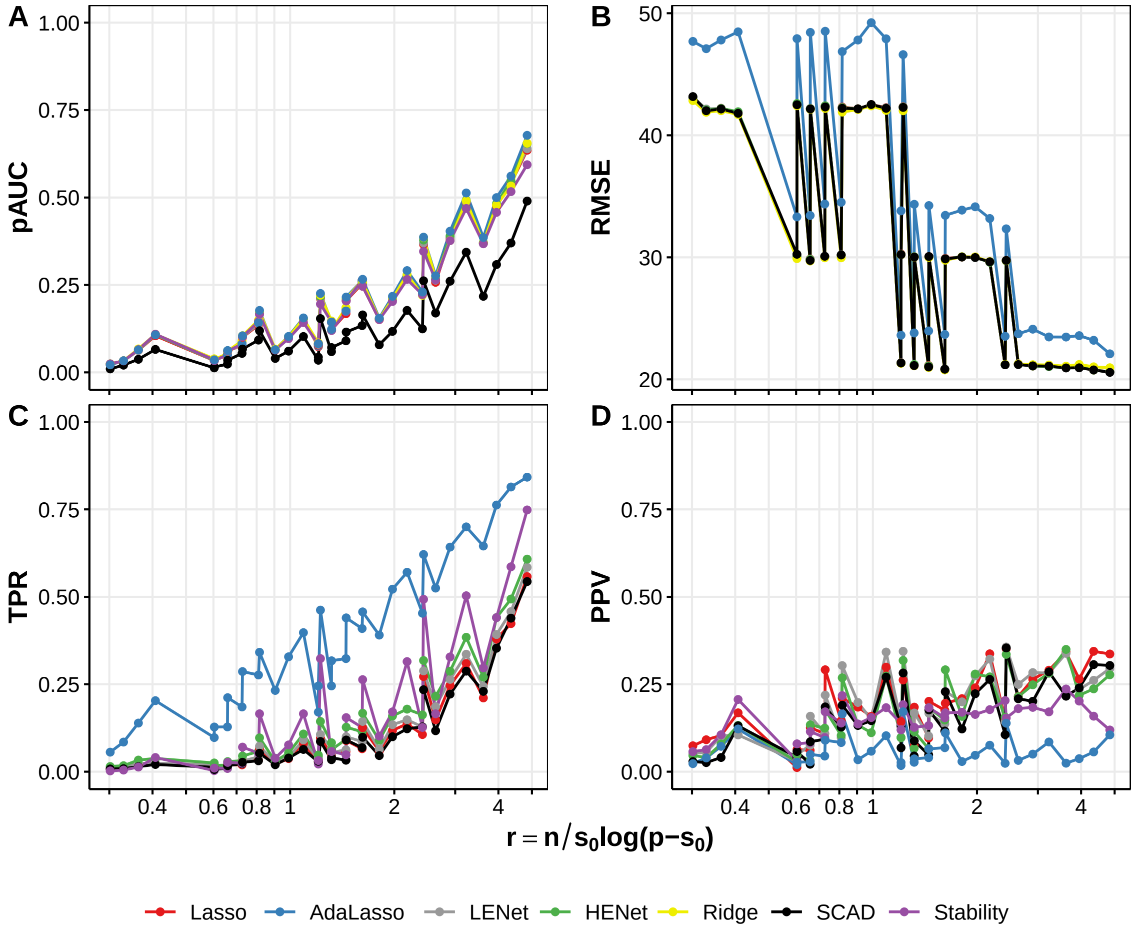

Figure S1: Ranking (A), prediction (B) and selection (C,D) performance versus the rescaled sample size $r = n/(s_0 log(p - s_0))$ for synthetic independence design scenarios. As Figure 1 in Main Text, but with SNR=0.5 (instead of SNR=2).

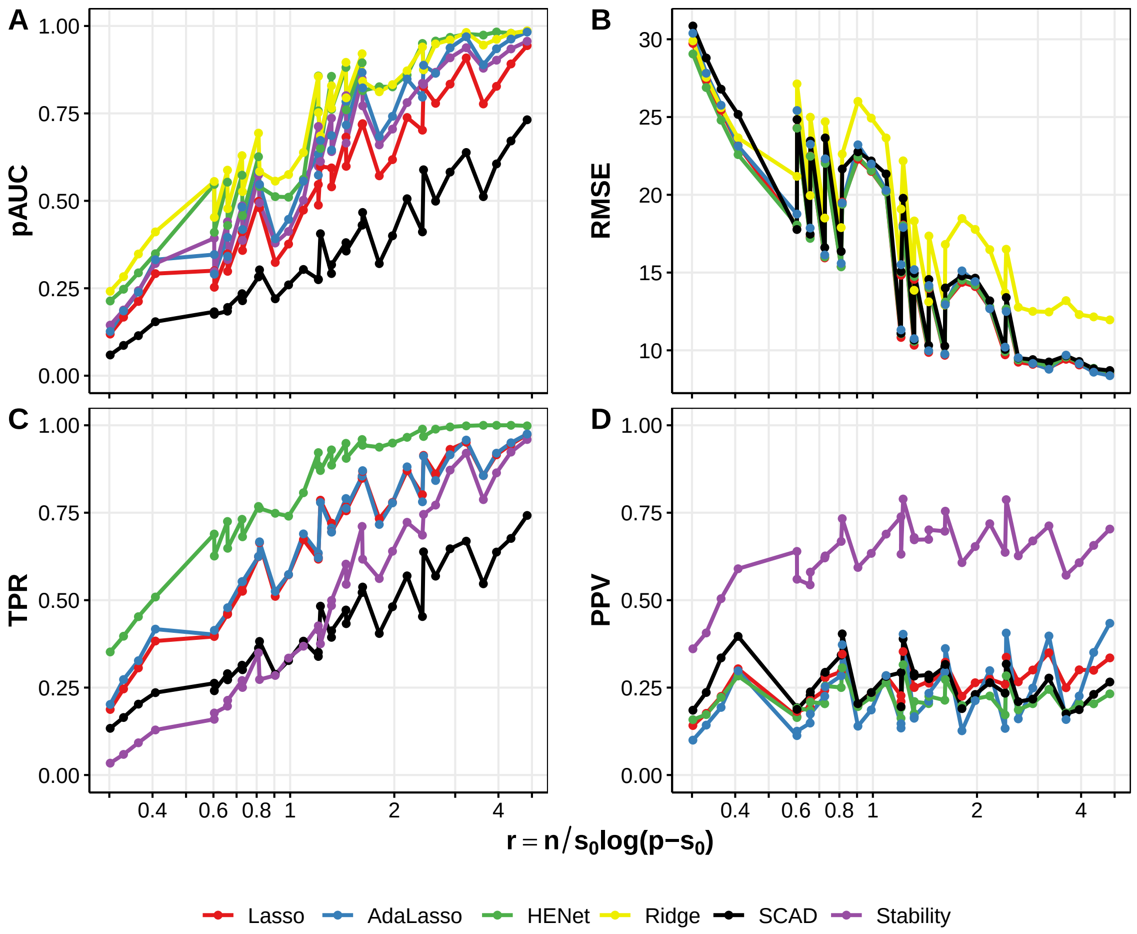

Figure S2: Ranking (A), prediction (B) and selection (C,D) performance versus the rescaled sample size $r = n/(s_0 log(p - s_0))$ for a semisynthetic "high"-correlation design scenario. As Figure 1 in Main Text, but for a semisynthetic "high"-correlation design with SNR=2 and $s_0^B = 5$.

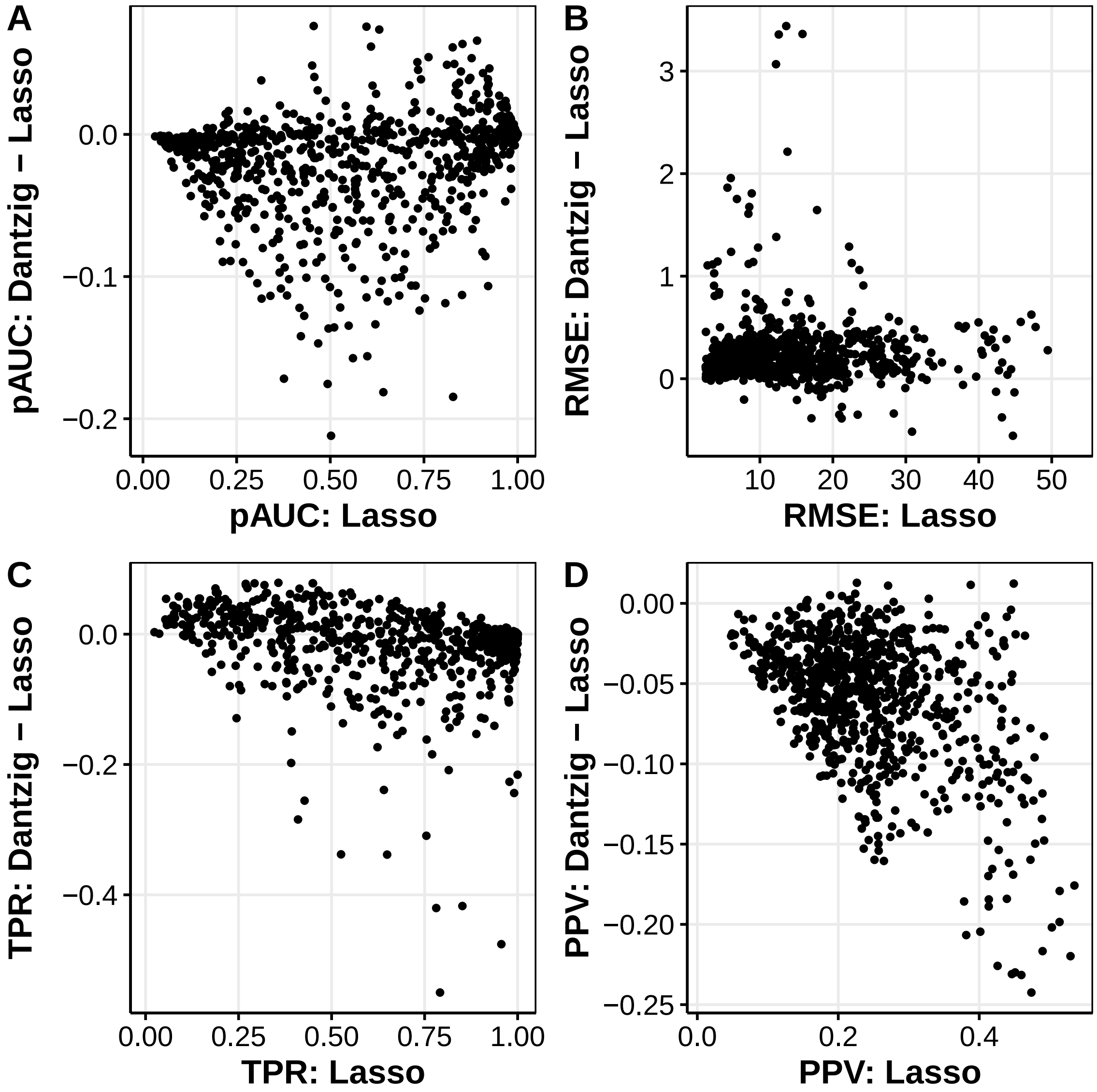

Figure S3: Difference in performance between Dantzig and Lasso (Dantzig - Lasso) versus Lasso performance for ranking (A), prediction (B) and selection (C,D) in synthetic data scenarios. Each point plotted represents a synthetic data scenario (both independence design and correlation design scenarios are plotted). For A, C and D, negative values on the y-axis indicate that Lasso is outperforming Dantzig. For B, a positive value indicates the same.

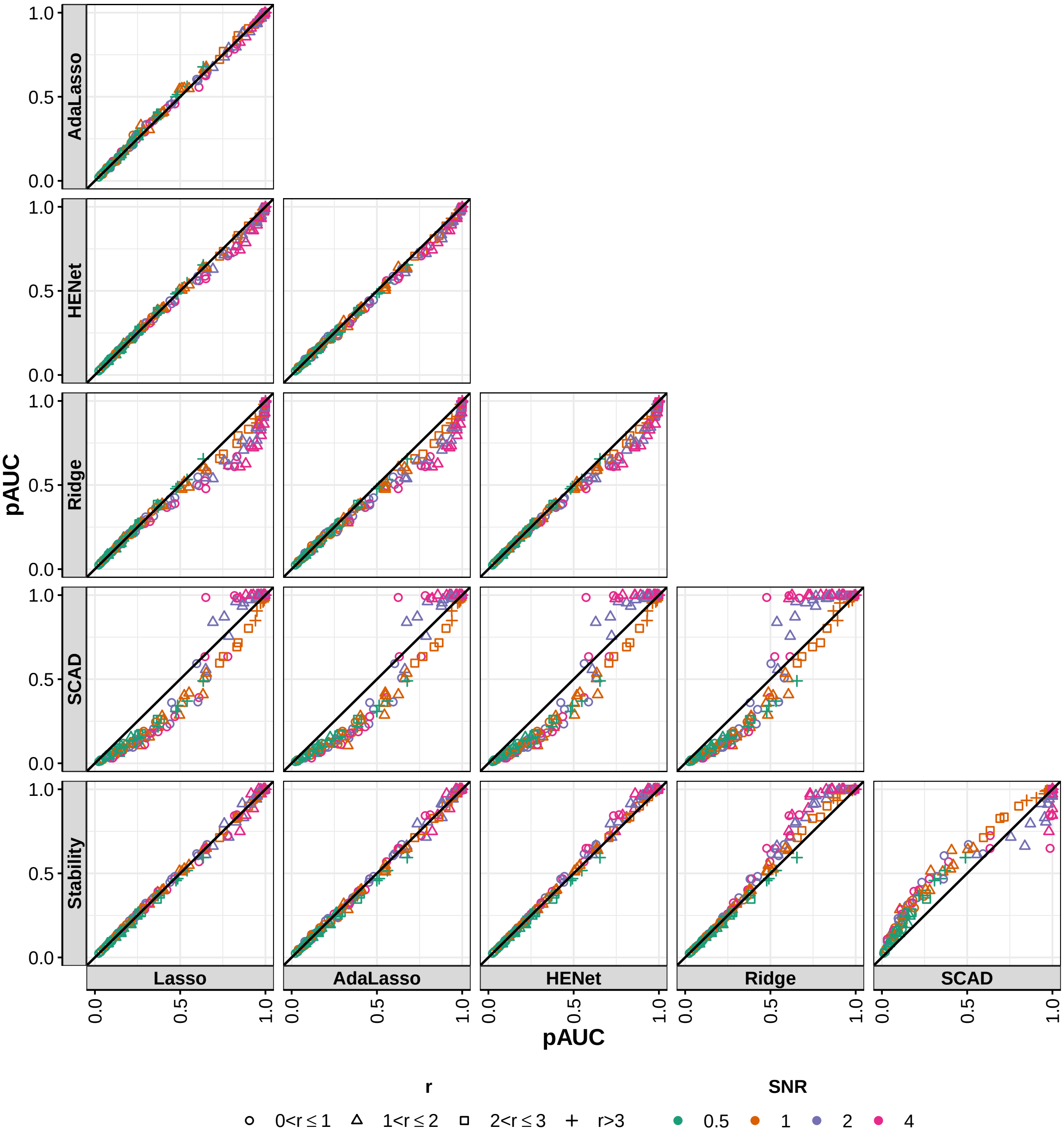

Figure S4: A comparison of method performance in synthetic independence design scenarios: ranking. Each panel plots the ranking performance of one method versus the ranking performance of another method. Each data point within a panel corresponds to an independence design scenario with color indicating SNR and symbol representing the value of the rescaled sample size r (categorized).

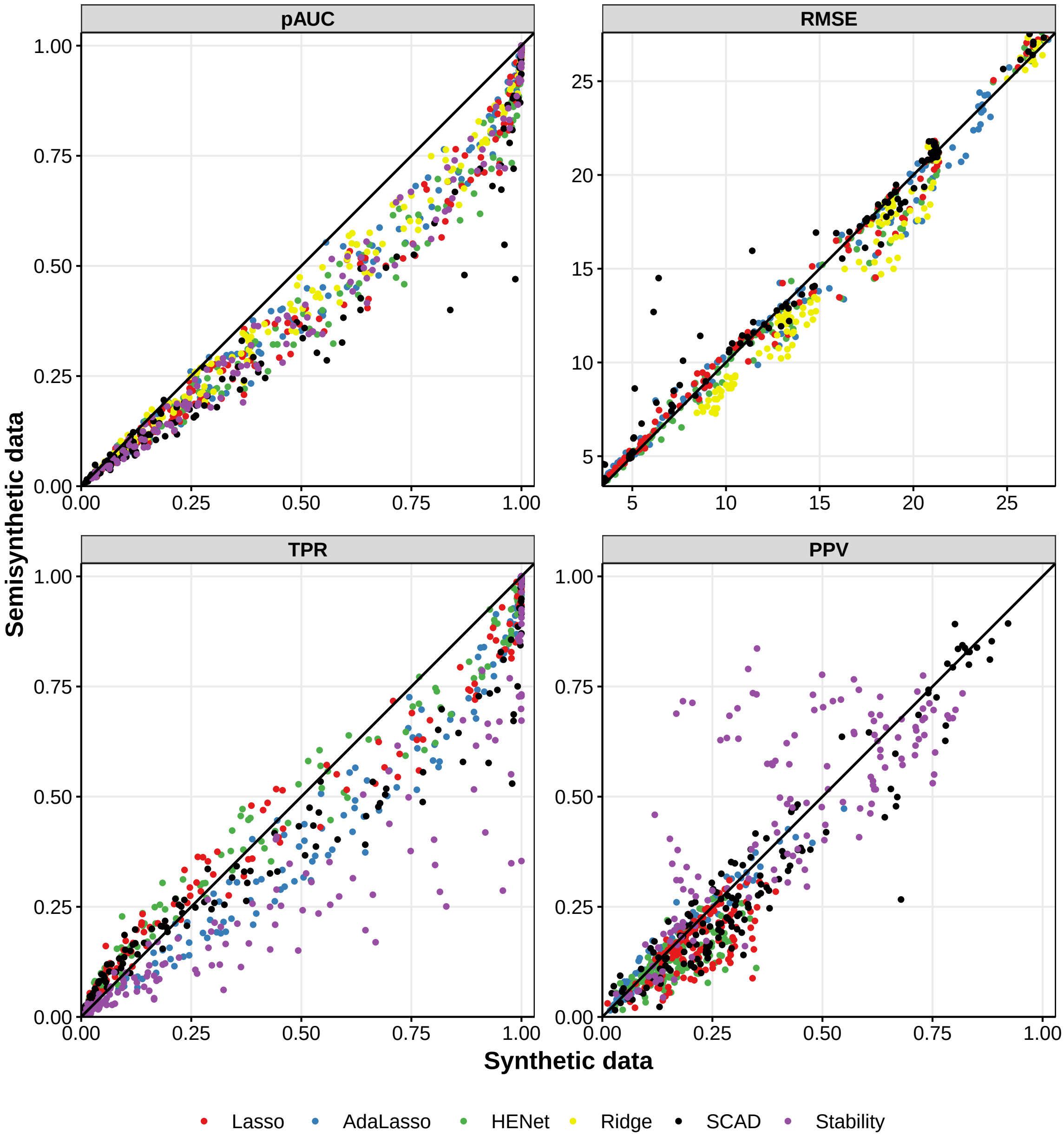

Figure S5: A comparison of method performance in the synthetic independence design and semisynthetic "low" correlation design. Each panel shows a different metric and each data point within a panel corresponds to a specific scenario (defined by n, p, s_0 and SNR), with color indicating method.

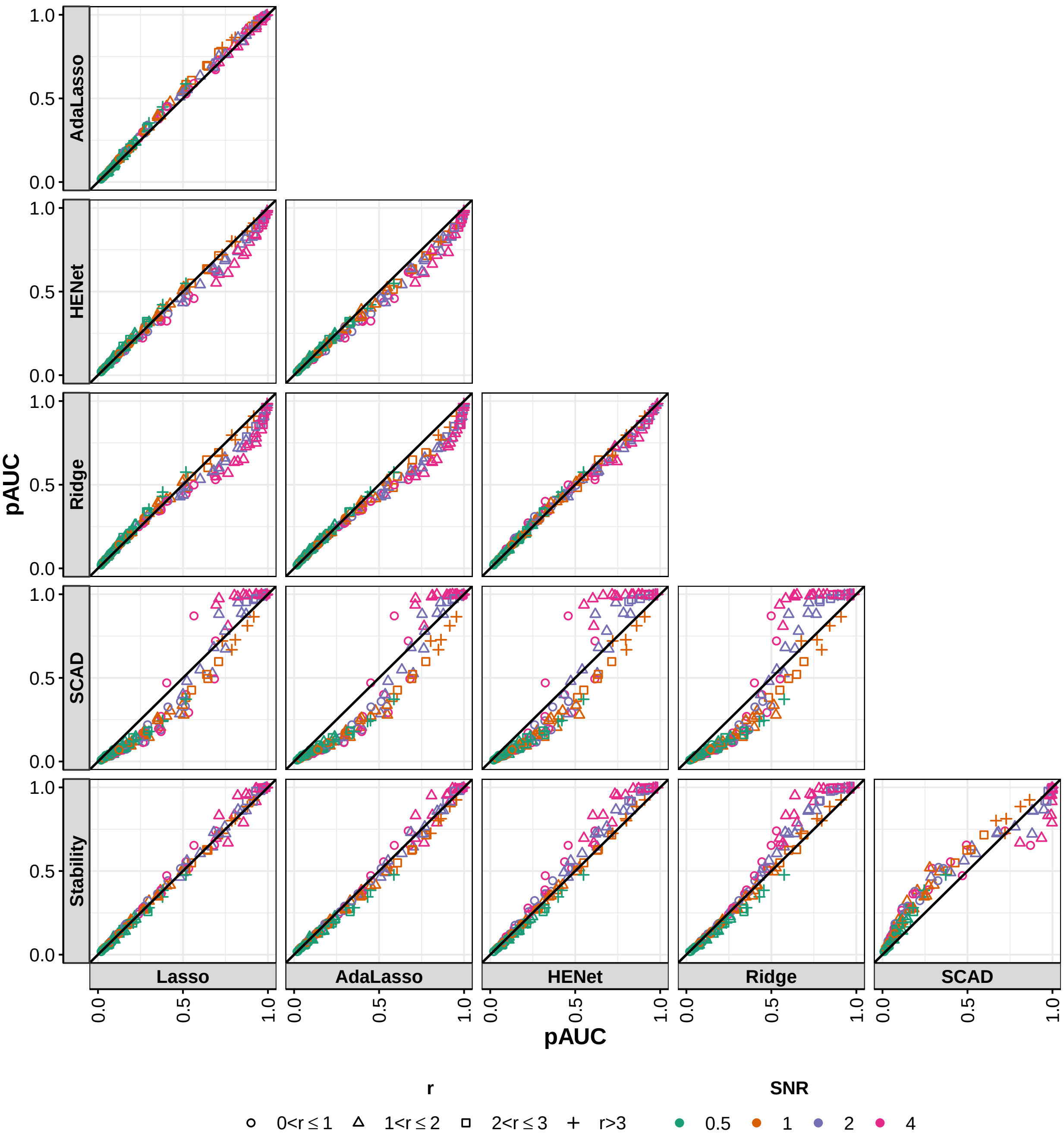

Figure S6: A comparison of method performance in semisynthetic "low"-correlation design scenarios: ranking. Each panel plots the ranking performance of one method versus the ranking performance of another method. Each data point within a panel corresponds to a scenario with color indicating SNR and symbol representing the value of the rescaled sample size r (categorized).

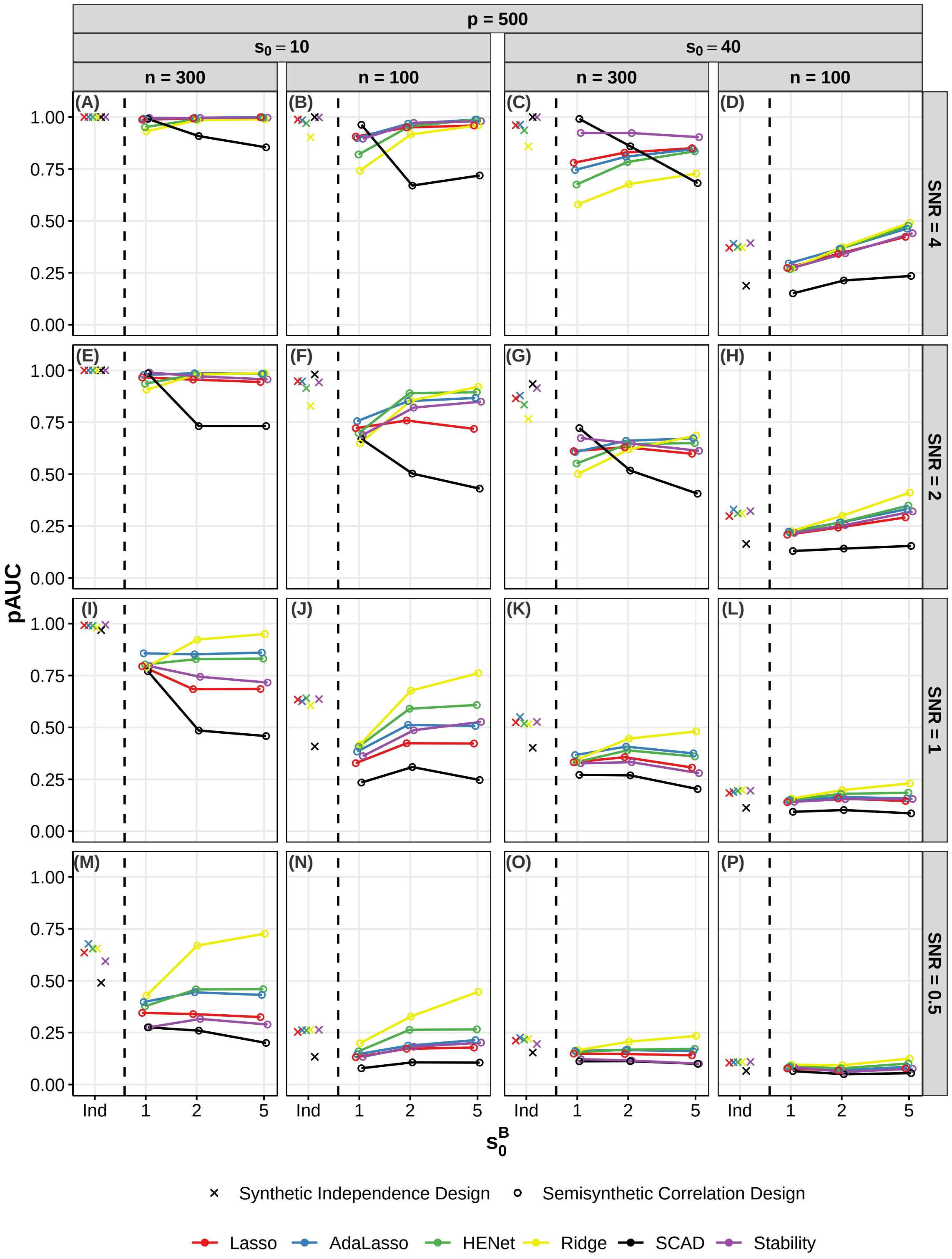

Figure S7: Ranking performance (pAUC) versus s_0^B (number of signals per block) for a subset of semisynthetic "high"-correlation designs. As Figure 3 in Main Text, but with p=500 (instead of p=2000) and all values of SNR are shown.

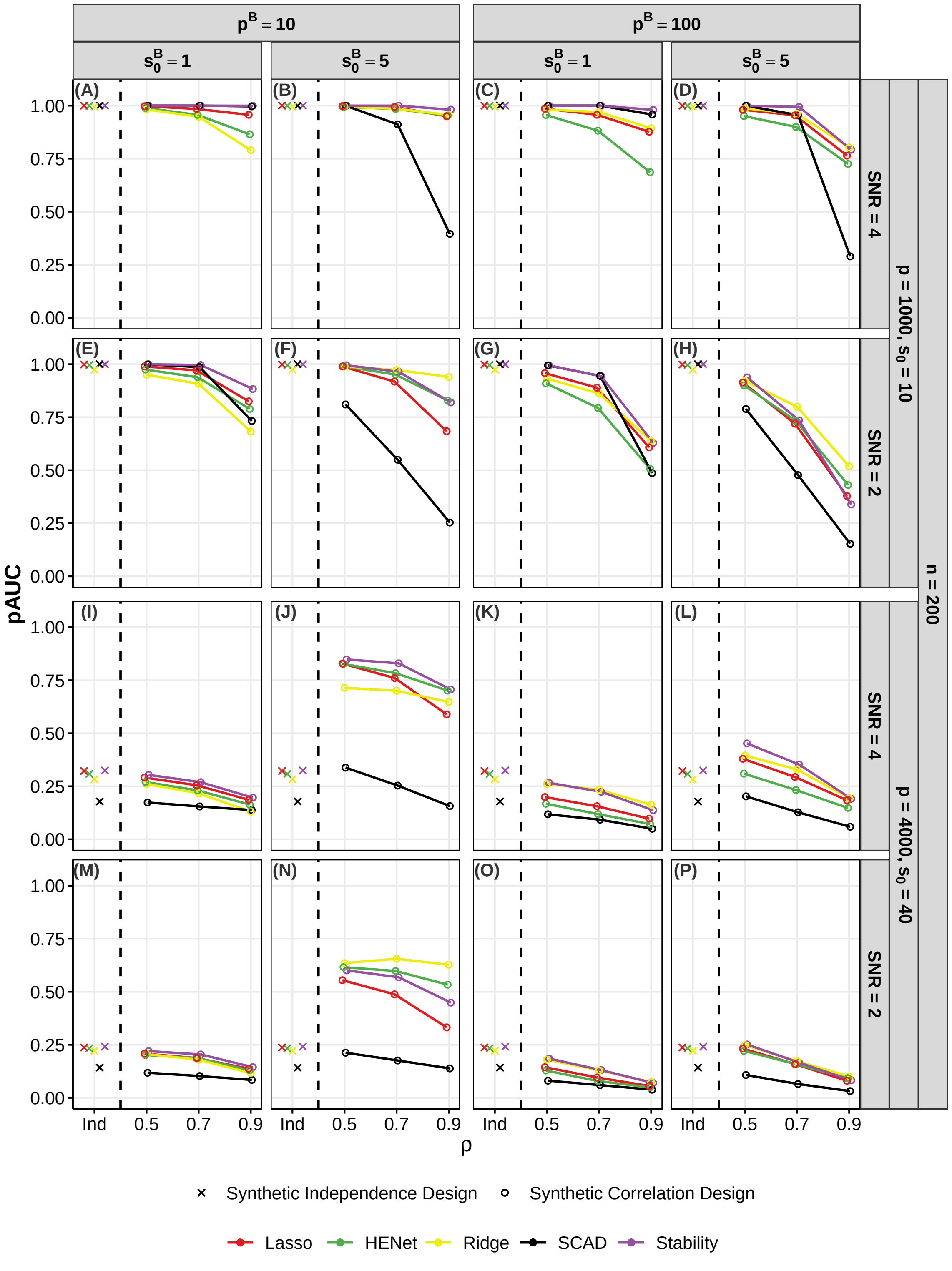

Figure S8: Ranking performance (pAUC) versus ρ (correlation strength) for a subset of synthetic pairwise correlation designs. As Figure 4 in Main Text, but with SNR=2 and 4 (instead of SNR=1).

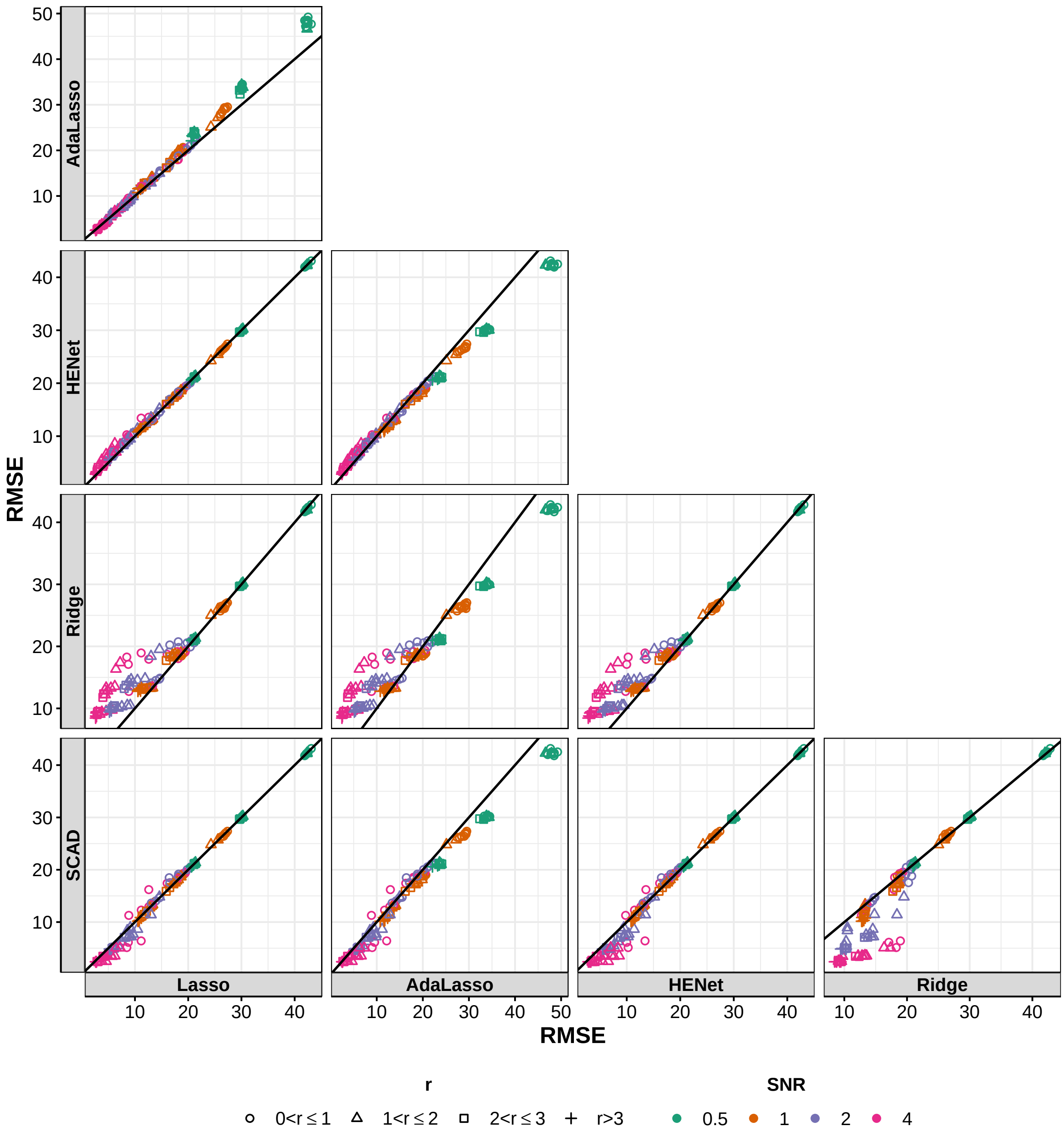

Figure S9: A comparison of method performance in synthetic independence design scenarios: prediction. Each panel plots the prediction performance of one method versus the prediction performance of another method. Each data point within a panel corresponds to an independence design scenario with color indicating SNR and symbol representing the value of the rescaled sample size r (categorized).

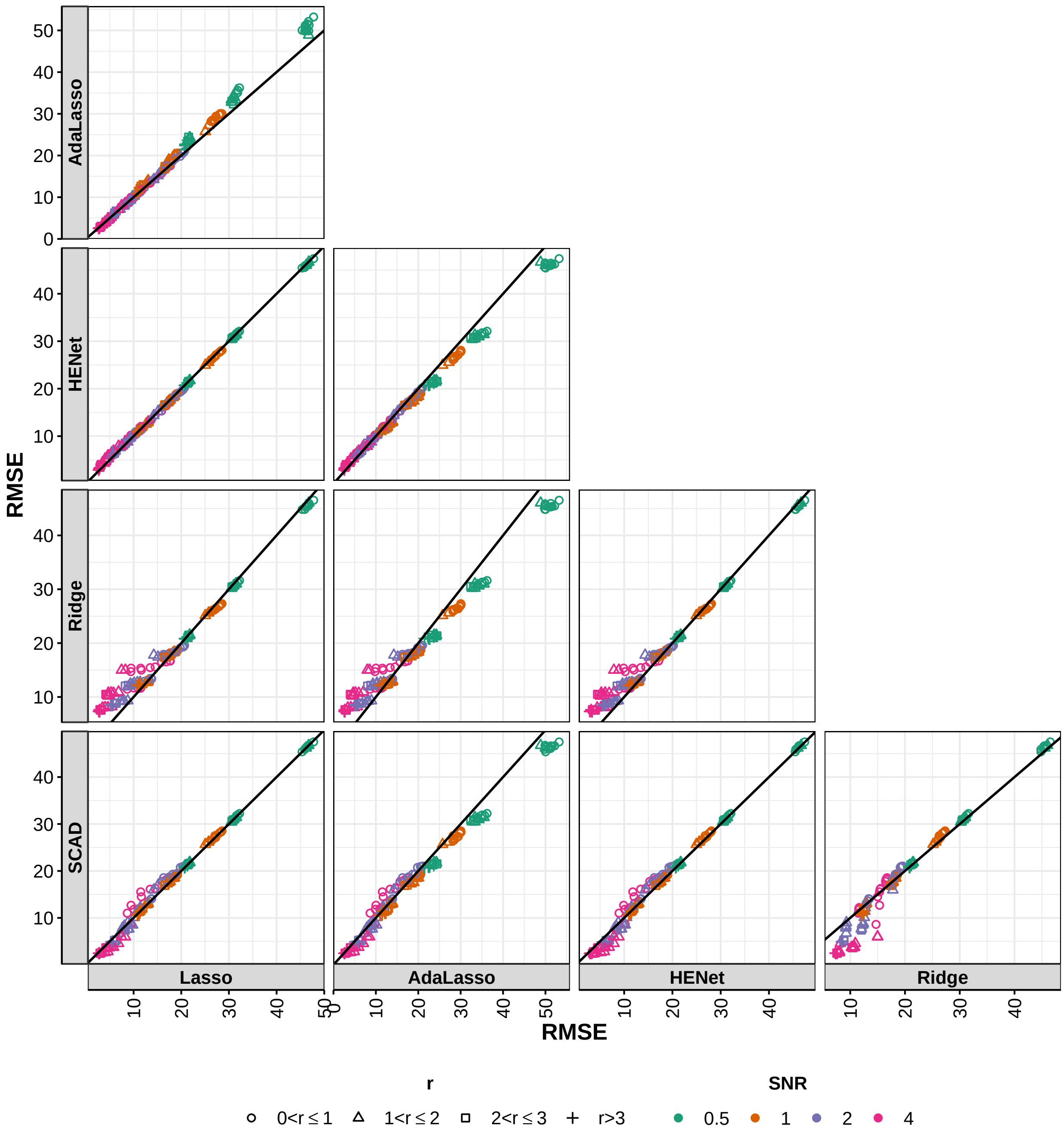

Figure S10: A comparison of method performance in semisynthetic "low"-correlation design scenarios: prediction. Each panel plots the prediction performance of one method versus the prediction performance of another method. Each data point within a panel corresponds to a scenario with color indicating SNR and symbol representing the value of the rescaled sample size r (categorized).

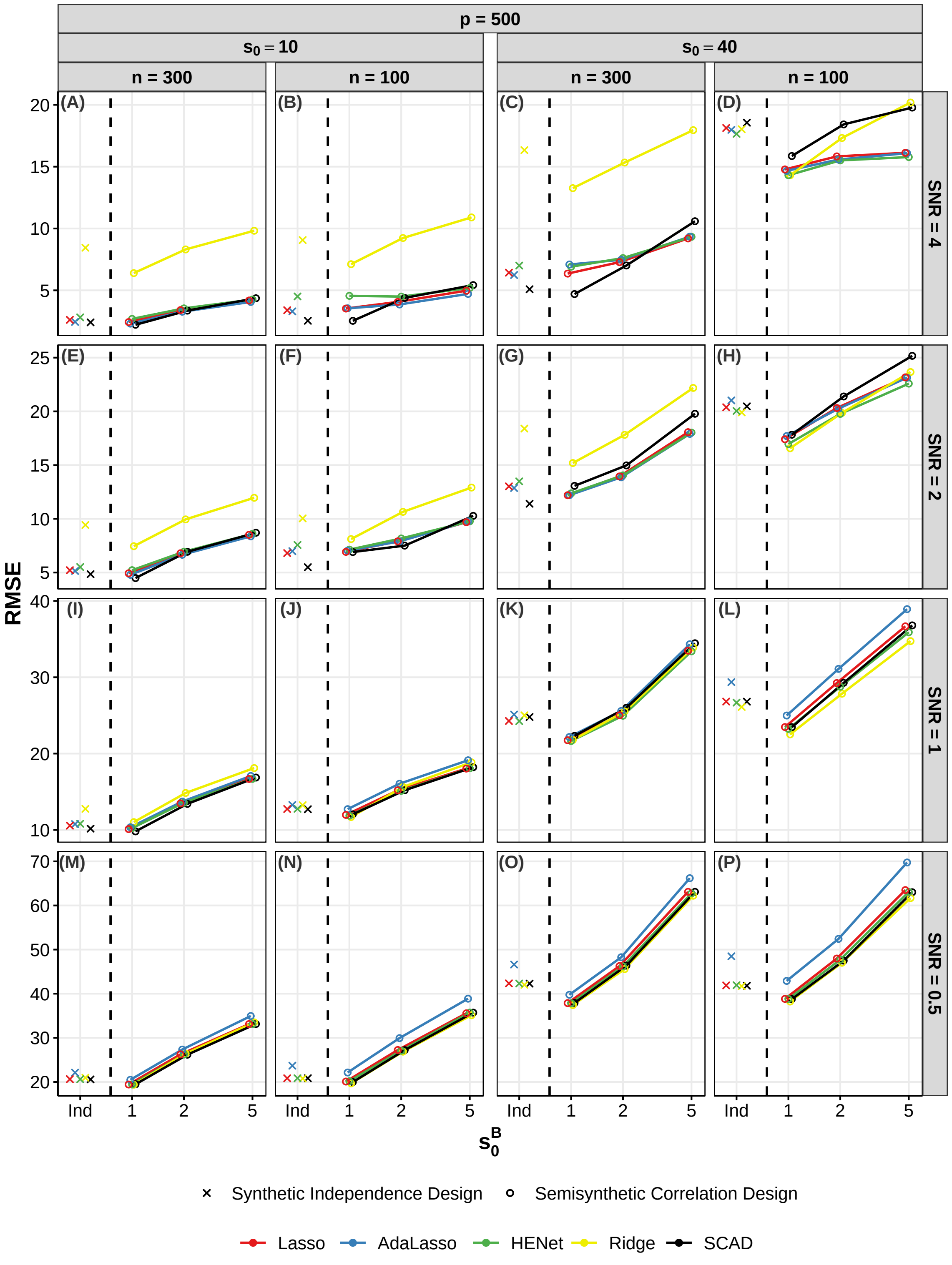

Figure S11: Prediction performance (RMSE) versus s_0^B (number of signals per block) for a subset of semisynthetic "high"-correlation designs. As Figure 6 in Main Text, but with p=500 (instead of p=2000) and all values of SNR are shown.

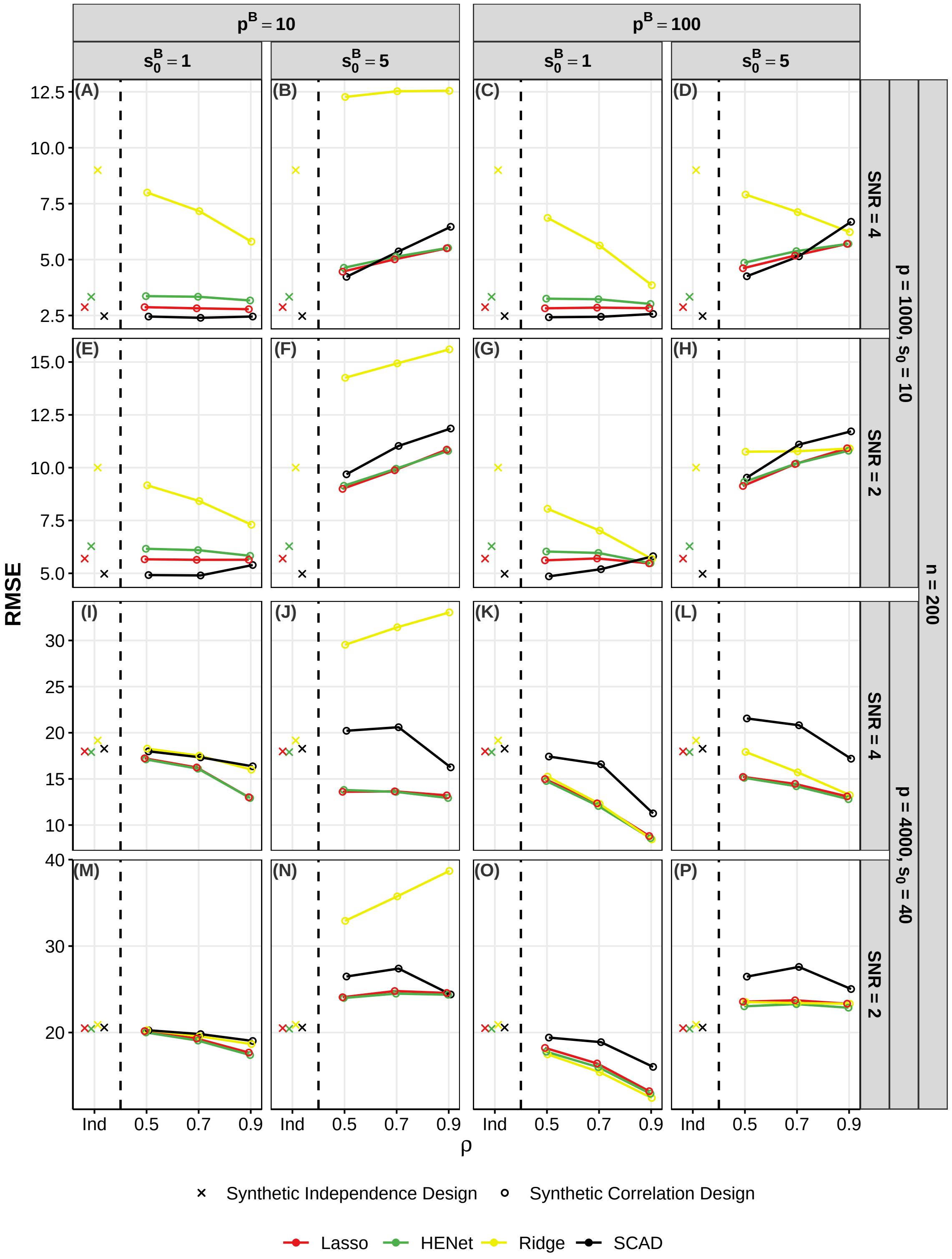

Figure S12: Prediction performance (RMSE) versus ρ (correlation strength) for a subset of synthetic pairwise correlation designs. As Figure 7 in Main Text, but with SNR=2 and 4 (instead of SNR=1).

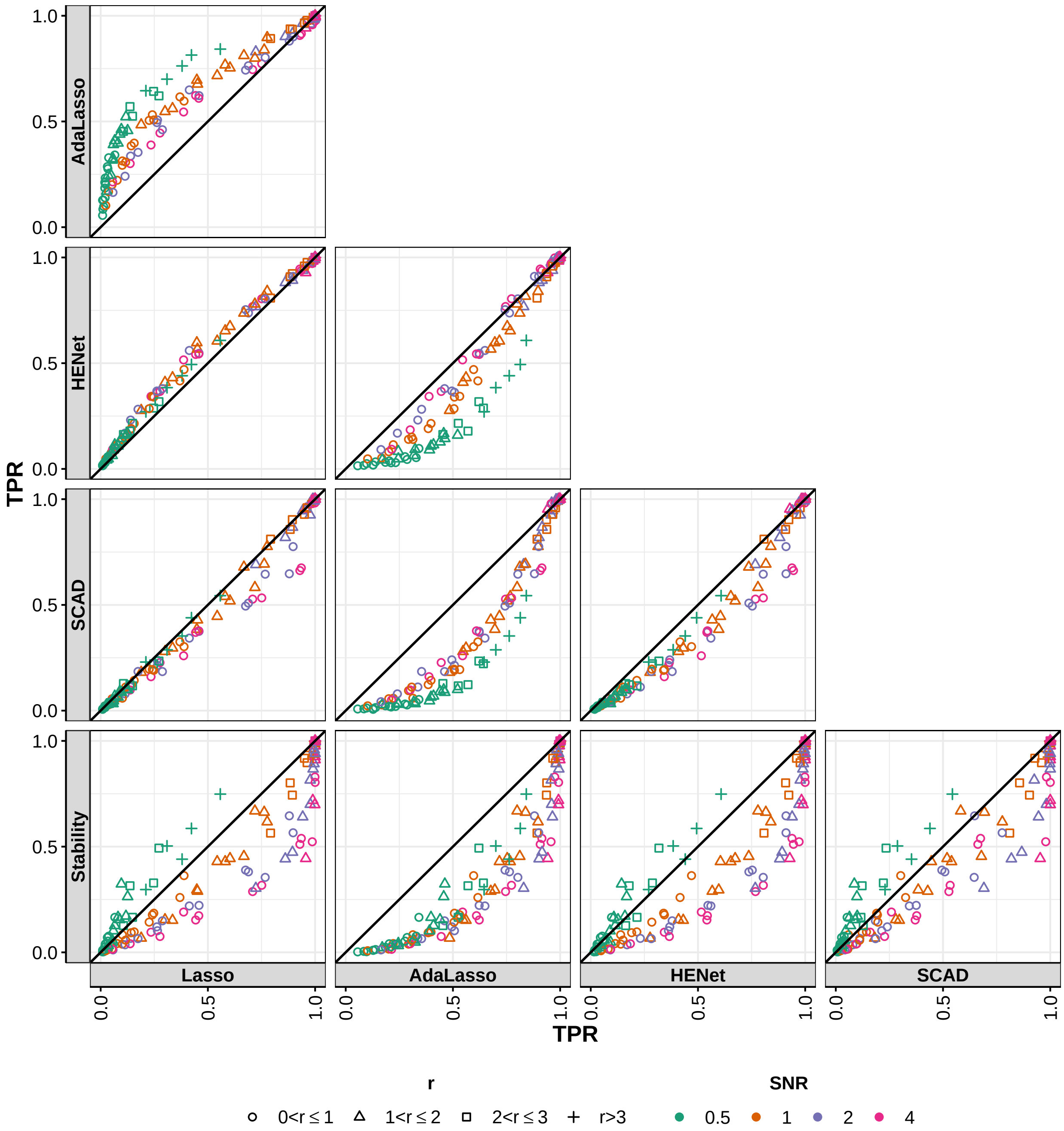

Figure S13: A comparison of method performance in synthetic independence design scenarios: selection - TPR. Each panel plots TPR of one method versus TPR of another method. Each data point within a panel corresponds to an independence design scenario with color indicating SNR and symbol representing the value of the rescaled sample size r (categorized).

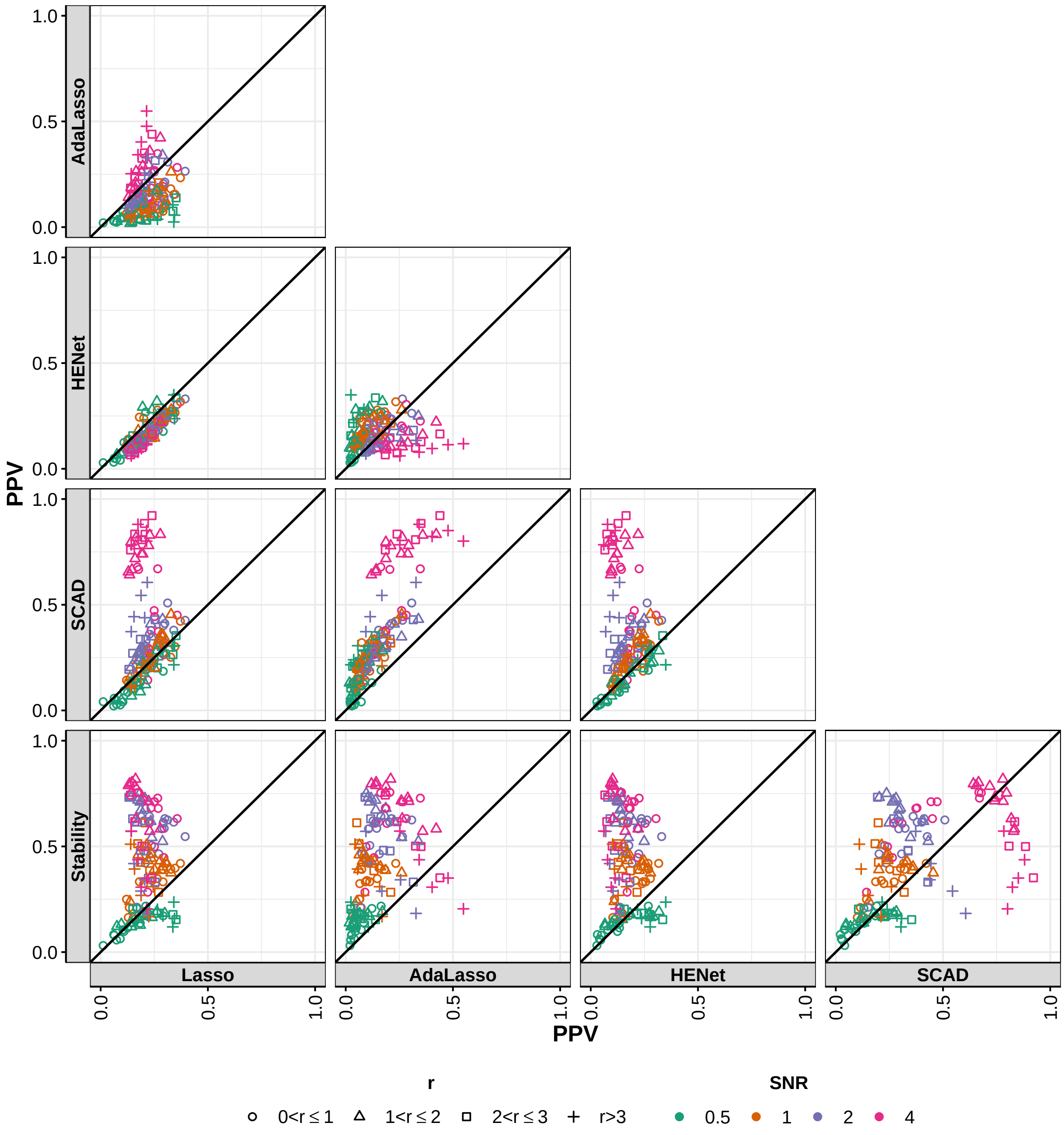

Figure S14: A comparison of method performance in synthetic independence design scenarios: selection - PPV. Each panel plots PPV of one method versus PPV of another method. Each data point within a panel corresponds to an independence design scenario with color indicating SNR and symbol representing the value of the rescaled sample size r (categorized).

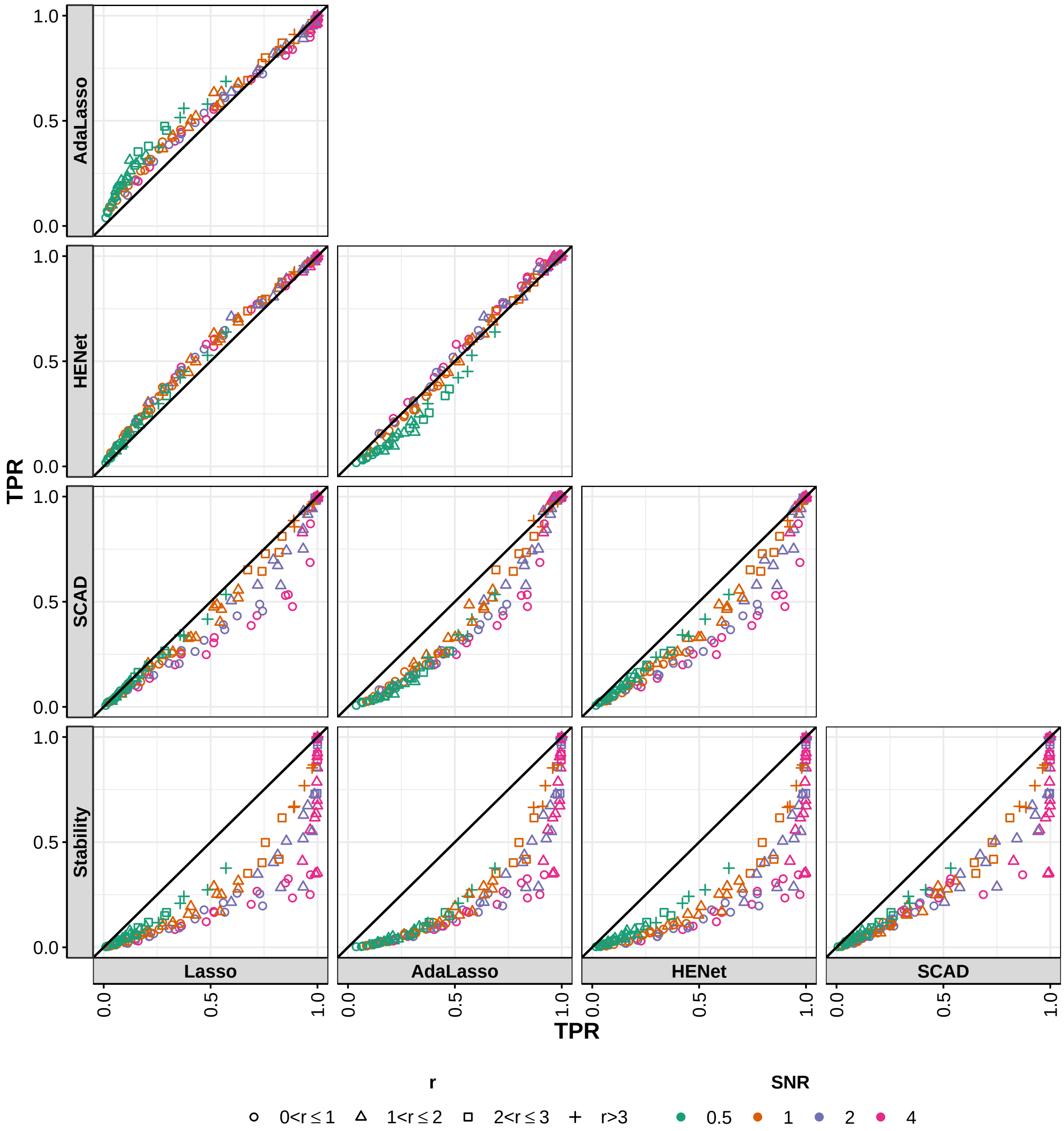

Figure S15: A comparison of method performance in semisynthetic "low"-correlation design scenarios: selection - TPR. Each panel plots TPR of one method versus TPR of another method. Each data point within a panel corresponds to a scenario with color indicating SNR and symbol representing the value of the rescaled sample size r (categorized).

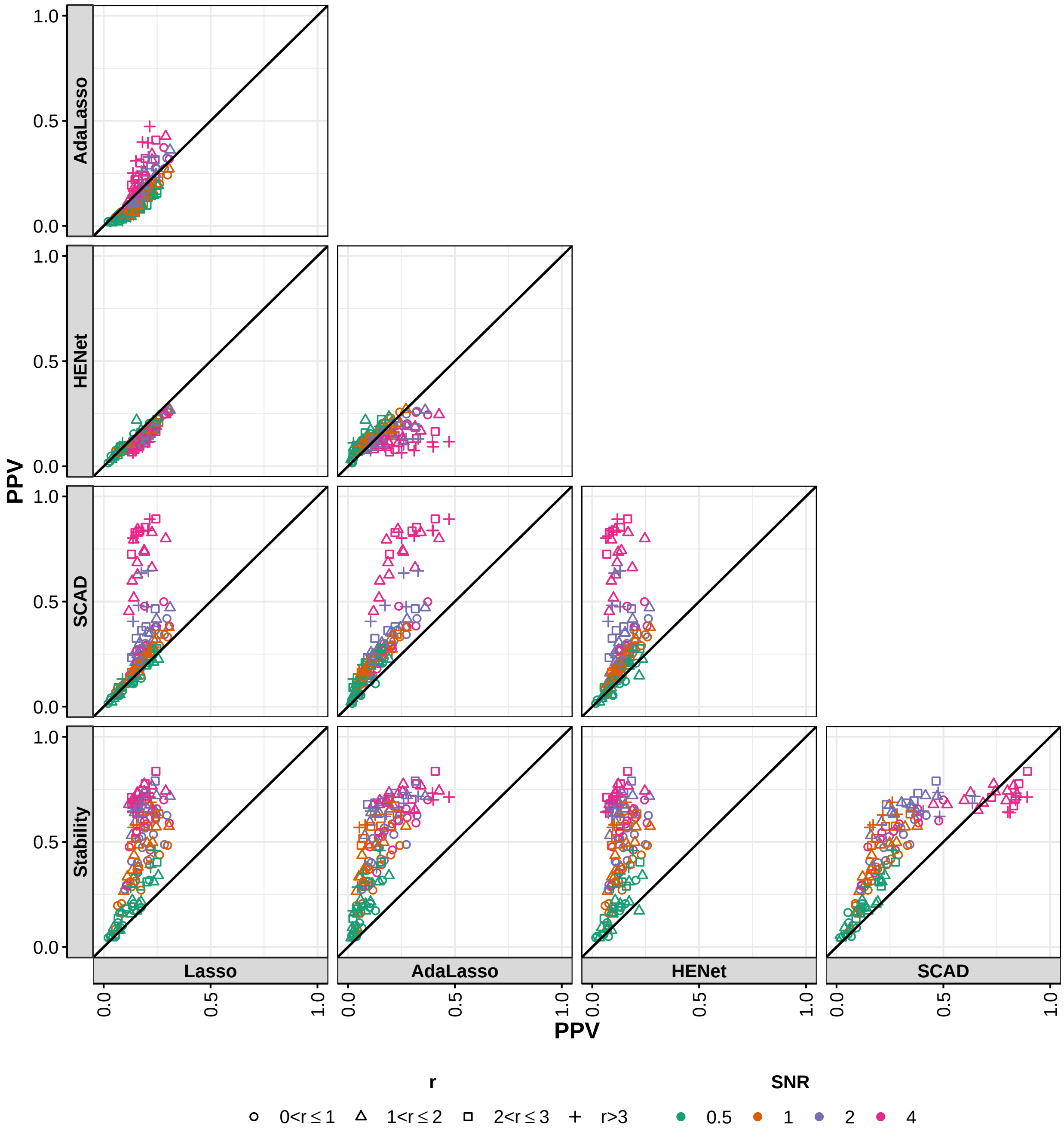

Figure S16: A comparison of method performance in semisynthetic "low"-correlation design scenarios: selection - PPV. Each panel plots PPV of one method versus PPV of another method. Each data point within a panel corresponds to a scenario with color indicating SNR and symbol representing the value of the rescaled sample size r (categorized).

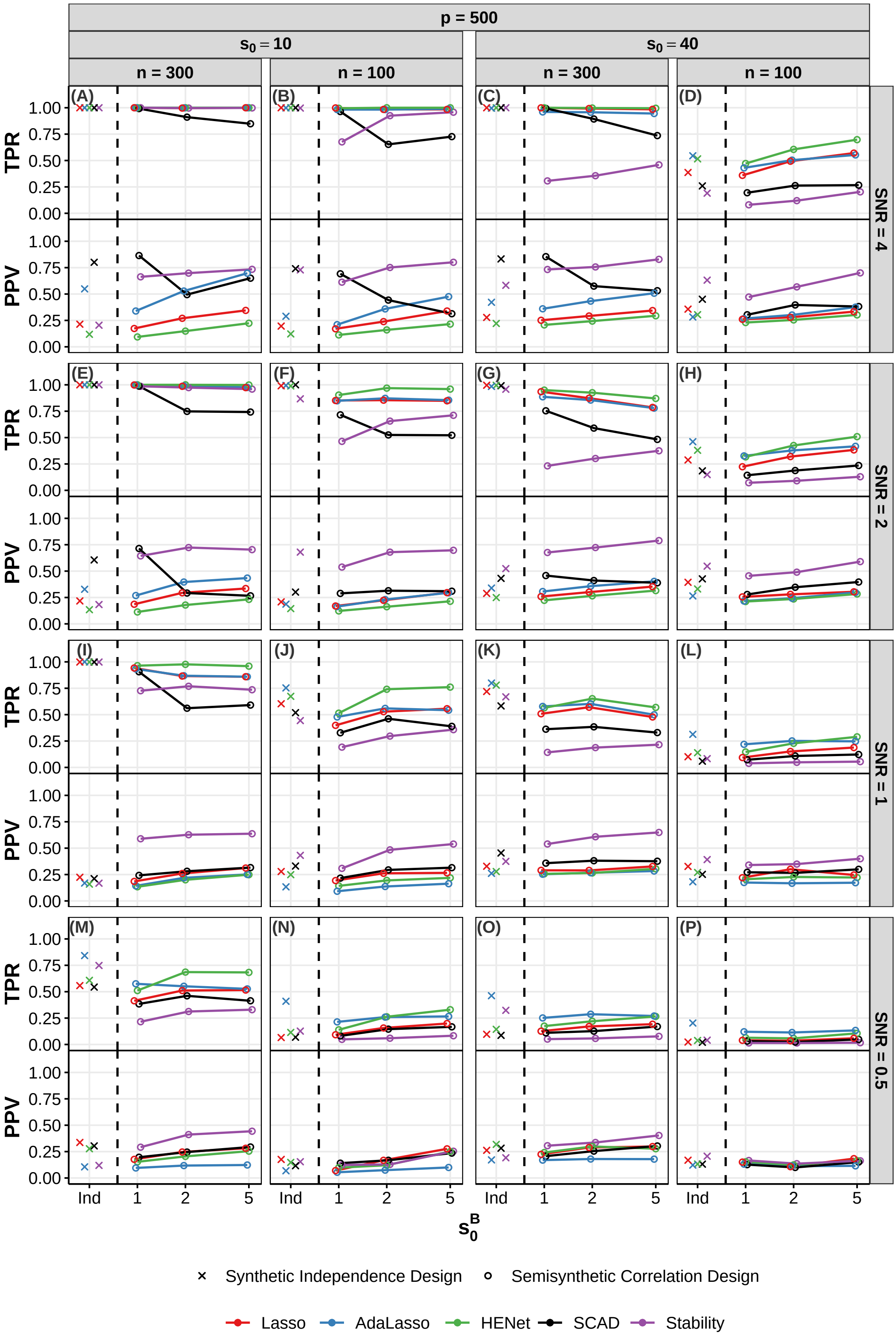

Figure S17: Selection performance (TPR and PPV) versus s_0^B (number of signals per block) for a subset of semisynthetic "high"-correlation designs. As Figure 9 in Main Text, but with p=500 (instead of p=2000) and all values of SNR are shown.

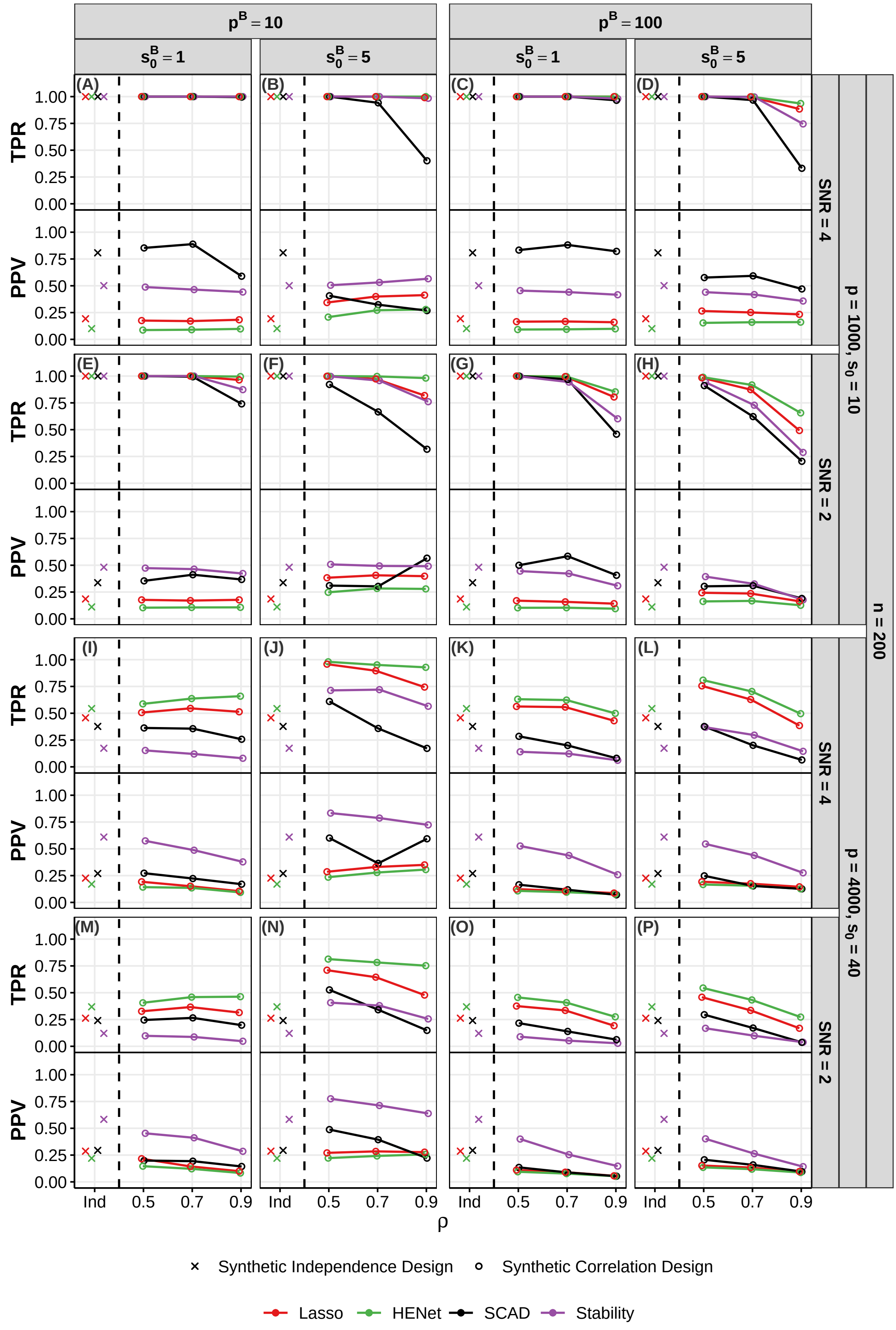

Figure S18: Selection performance (TPR and PPV) versus ρ (correlation strength) for a subset of synthetic pairwise correlation designs. As Figure 10 in Main Text, but with SNR=2 and 4 (instead of SNR=1).

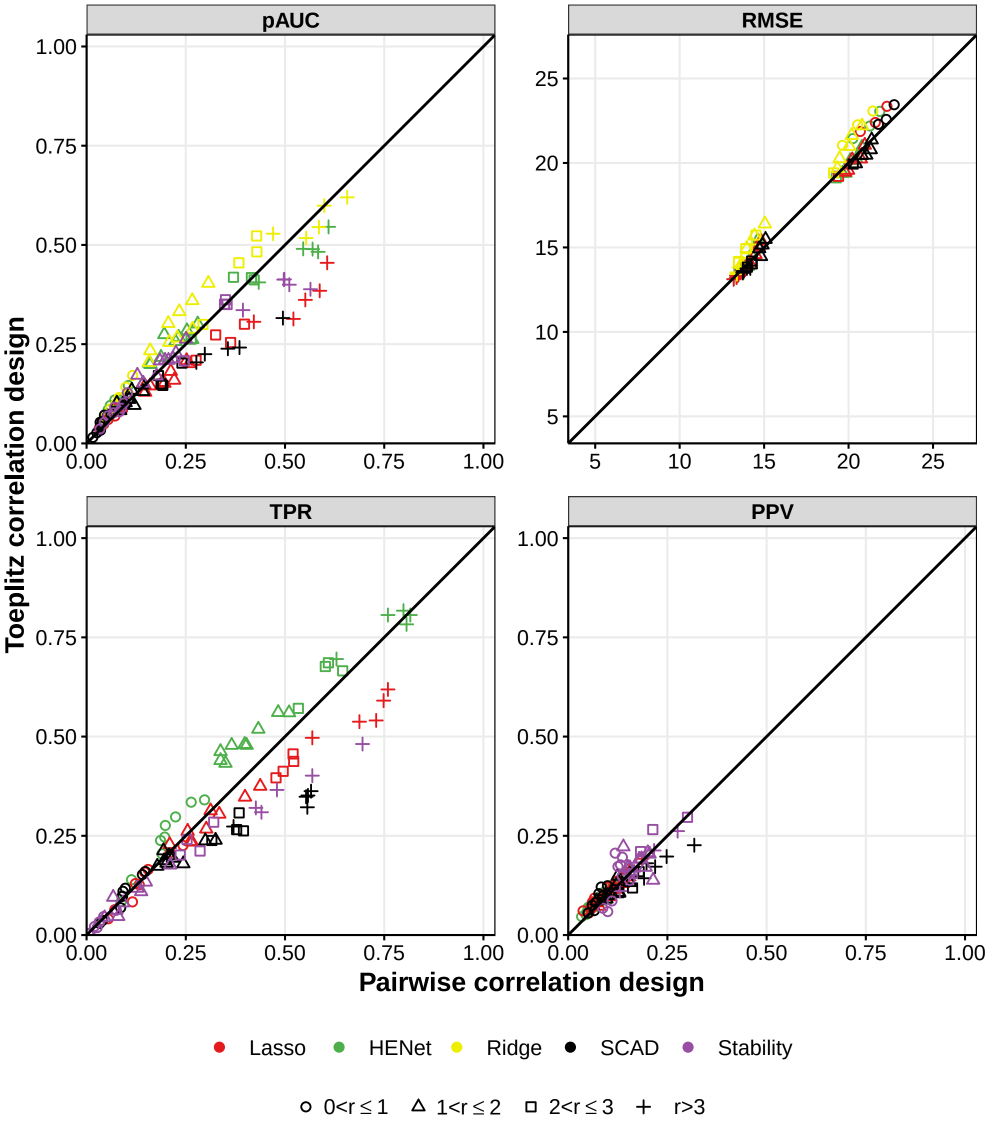

Figure S19: Comparison between Toeplitz correlation and pairwise correlation designs for ranking, prediction and selection performance. As Figure 11 in Main Text, but with SNR=1 (instead of SNR=2).

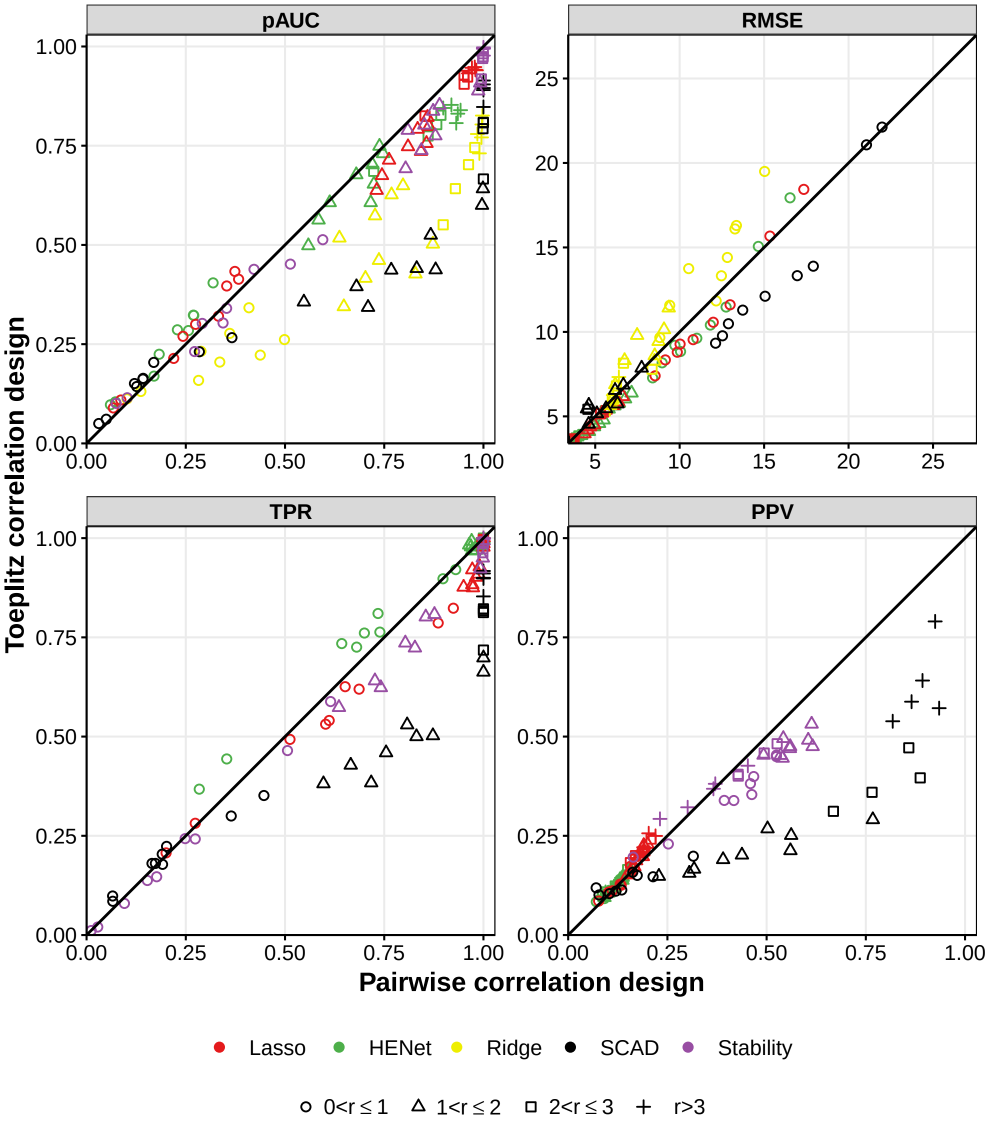

Figure S20: Comparison between Toeplitz correlation and pairwise correlation designs for ranking, prediction and selection performance. As Figure 11 in Main Text, but with SNR=4 (instead of SNR=2).

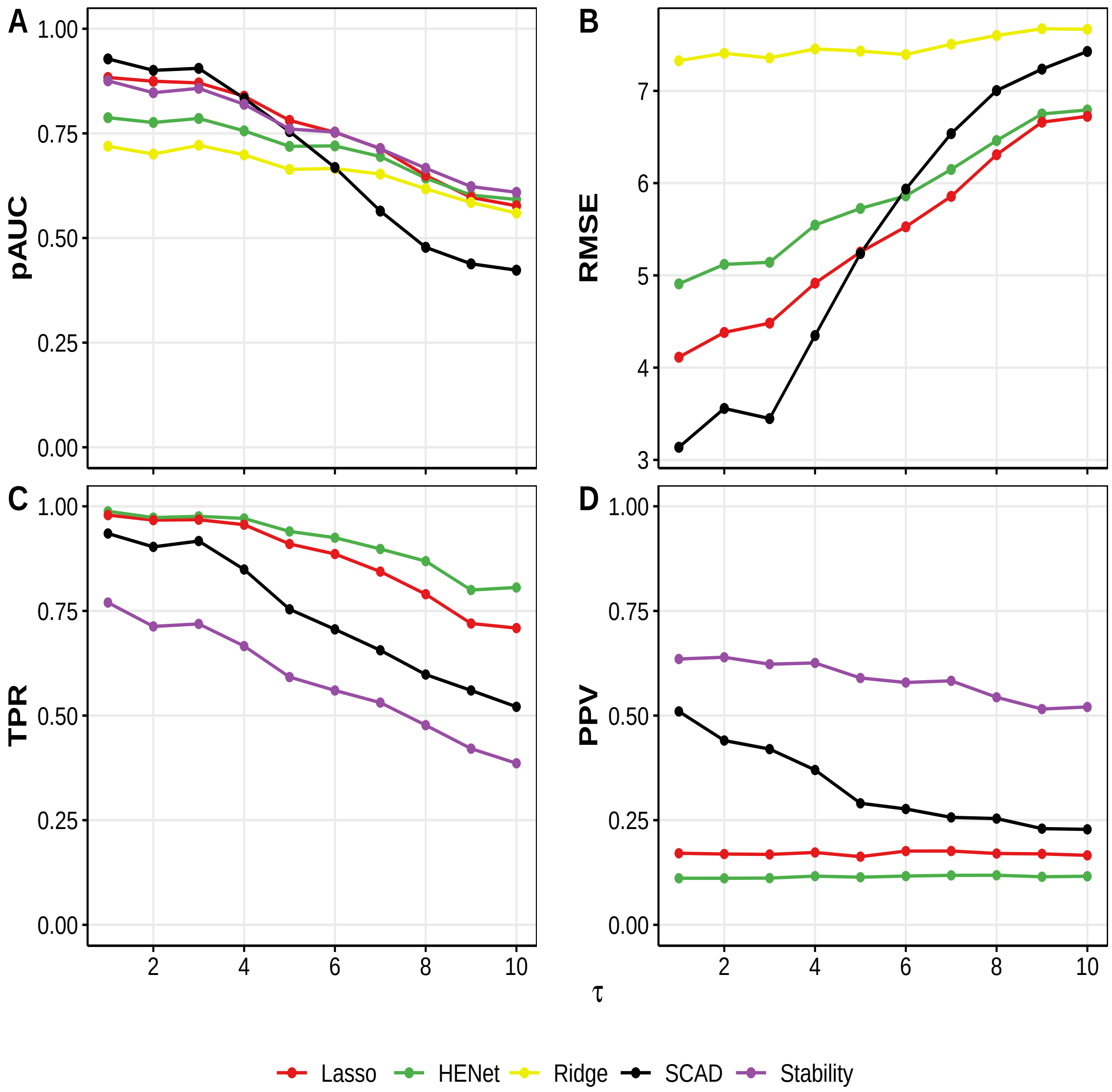

Figure S21: Semisynthetic (TCGA ovarian cancer expression) data analysis: "low"-correlation scenario with non-Gaussian error distribution. Semisynthetic training and test datasets were generated as described in the Main Text for the "low"-correlation scenario with $n = 100$, $p = 1000$ and $s_0 = 10$, but with 95% of error terms drawn from $N(0, \sigma^2)$ and the other 5% drawn from $N(0, (\tau\sigma)^2)$, with σ set such that SNR=4 and $\tau \in \{1, \ldots, 10\}$. $\tau = 1$ represents the standard set-up with noise drawn from a single Gaussian distribution. Ranking (A), prediction (B) and selection (C,D) performance are plotted against τ. Line color indicates method and results are averages over 100 replicates.
